# Development and internal and external validation of a nomogram model for frailty risk among hospitalised older people using comprehensive geriatric assessment data

**DOI:** 10.1186/s12877-023-04426-8

**Published:** 2023-11-02

**Authors:** Hong Lyu, Wenhui Jiang

**Affiliations:** 1 Xi’an Jiaotong University Health Science Center, Xi’an, 710061 Shaanxi Province China; 2 Outpatient department of Geriatrics, Shandong Provincial Hospital Affiliated to Shandong First Medical University, Jinan, 250021 Shandong Province China

**Keywords:** Frailty, Frailty prediction, Frailty prevention, Frailty Nomogram prediction model, Comprehensive geriatric assessment

## Abstract

**Background:**

Currently, there are few such studies about establishing the frailty prediction model on the basis of the research on the factors influencing frailty in older patients, which can better predict frailty and identify its risk factors, and then guide the formulation of intervention measures precisely, especially in the hospital setting in China. Meanwhile, comprehensive geriatric assessment (CGA) can provide measurable and substantial health improvements for frail older people. The study aimed to develop a nomogram model for frailty risk among hospitalised older people using CGA data and validated its predictive performance for providing a basis for medical staff to grasp the risk and risk factors of older inpatients’ frailty conveniently and accurately, and to formulate reasonable nursing intervention plan.

**Methods:**

We used CGA data of individuals over age 64. Demographic characteristics, geriatric syndrome assessment, and frailty assessment based on the FRAIL scale were included as potential predictors. Significant variables in univariate analysis were used to construct risk models by logistic regression analysis. We used the root mean square (rms) to develop the nomogram prediction model for frailty based on independent clinical factors. Nomogram performance was internally validated with Bootstrap resampling. The final model was externally validated using an independent validation data set and was assessed for discrimination and calibration.

**Results:**

Data from 2226 eligible older inpatients were extracted. Five hundred sixty-two older inpatients (25.25%) suffered from frailty. The final prediction model included damaged skin, MNA-SF, GDS-15, Morse risk scores, hospital admission, ICI-Q-SF, Braden score, MMSE, BI scores, and Caprini scores. The prediction model displayed fair discrimination. The calibration curve demonstrated that the probabilities of frailty predicted by the nomogram were satisfactorily matched.

**Conclusions:**

The prediction model to identify hospitalised older people at high risk for frailty using comprehensive geriatric assessment data displayed fair discrimination and good predictive calibration. Therefore, it is inexpensive, easily applied, and accessible in clinical practice, containing variables routinely collected and readily available through consultation. It will be valuable for grasp older inpatients at high risk of frailty and risk factors in hospital setting to guide the formulation of intervention measures precisely for reversing and preventing frailty.

## Background

Frailty is the most problematic aspect of population ageing. Older people are vulnerable to external stress because physiological reserves decline with age, which can cause adverse health outcomes such as falls, disability, fractures, and death [1, 2]. Frailty prevalence increases with age in those 65 and over, ranging from 7 to 16.3% [3]. The prevalence of Frailty in people over 65 years of age in Europe ranges from 5.8–27.3% [4]. For those aged 80 to 89, it’s about 20%, and for those over 90, it’s 33.3% [5]. 18.7% of China’s population is over the age of 60 in 2020, an increase of 5.44% from 10 years ago [6]. Frailty leads to a loss of independence and therefore poses great challenges for families, carers and society. Accurately focusing on the care needs of the older people, improving their self-care ability, quality of life, and preventing frailty will be the main geriatric care goals in China and the world in the few decades.

Frailty due to aging is avoidable. We can manage and prevent it to promote a healthier and longer life. Preventative interventions and frailty reversal will be possible by early screening and timely intervention for its risk factors.

Many frailty intervention programs have proven effective in preventing, delaying, or reversing frailty [7–9].

A critical step in prevention is identifying older people who are at increased risk and the risk factors to manage frailty early. The Asia-Pacific Clinical Practice Guidelines for the Management of Frailty strongly recommend that a validated measurement tool be used to identify frailty [10]. Several models and scales have been proposed to detect frailty [11–13]. Previous studies have shown some of the risk factors for frailty in old people. A Korea cohort study with 3011 participants aged 70 to 84 in the local communities revealed that depression, ADL, worse physical function, lower balance confidence, Mini Nutritional Assessment had a significant effect on frailty [14]. Another international systematic review of longitudinal studies among community-dwelling older adults showed that older age, obesity, depressive symptoms were longitudinal associated with frailty.

However, there are few such studies about establishing the frailty prediction model on the basis of the research on the factors influencing frailty in older patients, which can better predict frailty and identify its risk factors, and then guide the formulation of intervention measures precisely, especially in the hospital setting in China. Therefore, it has a limited predictive value in identifying hospitalised older people at risk [15].

As one of the ancient mathematical models, the nomogram model has been widely used in risk assessment of clinical diseases because it can intuitively calculate the approximate probability of clinical adverse events according to the value of predictive variables in the graph [16].

Comprehensive geriatric assessment (CGA), covering a comprehensive assessment of the physiological, psychological, spiritual, and social support and needs of the older people, which can provide measurable and substantial health improvements for frail older adults, including increased independence and reduced mortality [17]. For these reasons, we addressed the limitations described above using CGA data collected in hospital settings. Therefore, in order to provide a basis for medical staff to grasp the risk and risk factors of older inpatients’ frailty conveniently and accurately, and to formulate reasonable nursing intervention plan, this study aimed to develop a nomogram model for frailty risk among hospitalised older people using CGA data and to internally and externally validate its predictive performance.

## Methods

### Participants

We estimated the sample size based on multiple logistic regression analysis [18]. Literature reviews identified approximately 11 variables that contribute to frailty [19]. The number of frailty patients should be proportionately 10 times than those of risk factors [20]. Based on a previous study [21], the prevalence of inpatient frailty was 50%. A minimum sample size of 220 was established.

**Fig. 1:**
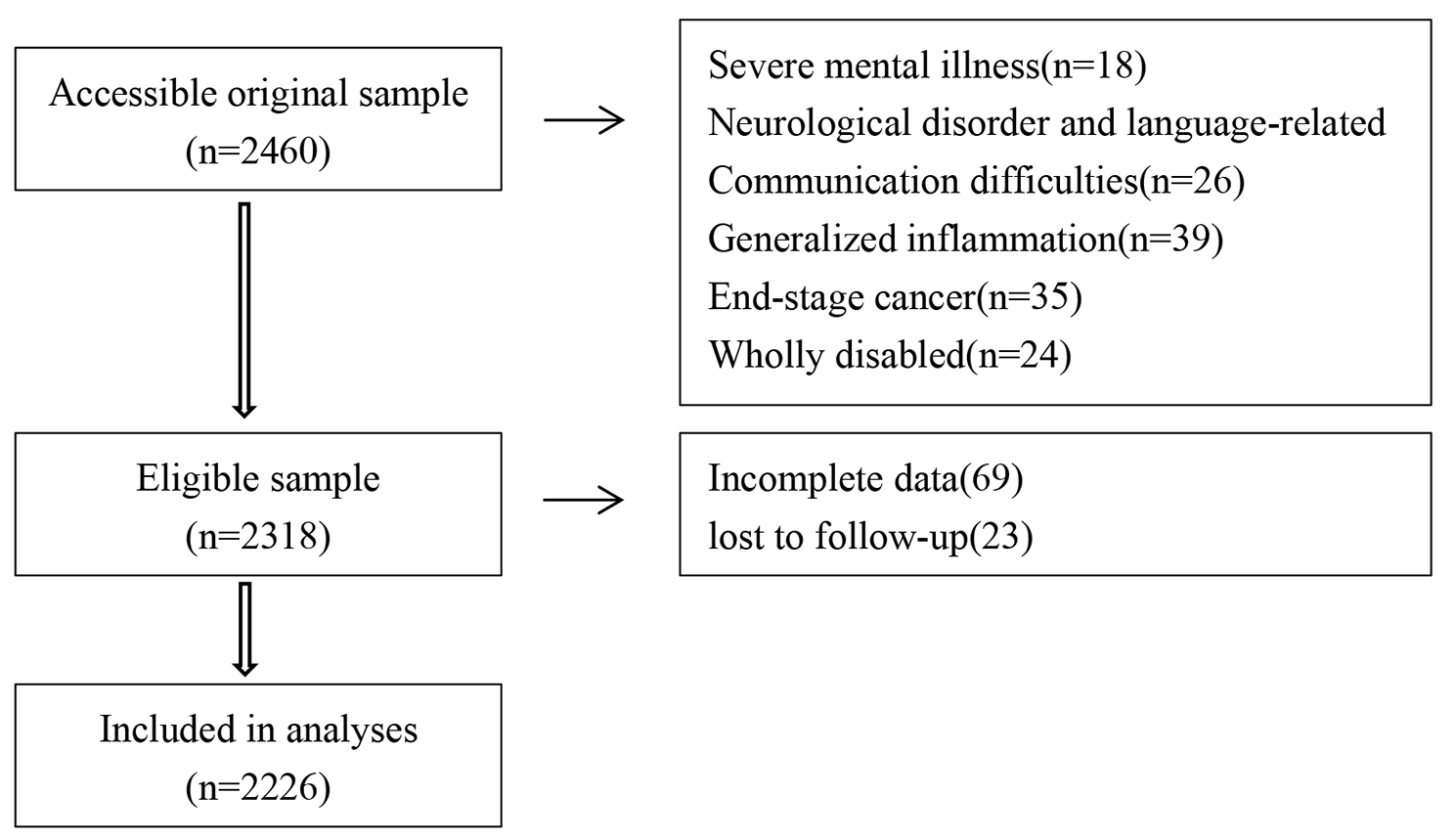
Flow chart of participants recruitment in study of Nomogram Model for Frailty Risk

Prior to modelling, the dataset was divided into modelling and validation sets. A total of 2226 older inpatients in the Department of Geriatrics of a tetiary grade A hospital in Shandong Province were selected by convenience sampling. Of these patients, 1481 (66.53%) admitted from January 2019 to December 2020 were the modelling set, and 745 (33.47%) were admitted from January to December 2021 were the validation set. Figure 1 showed the sample screening process.

### Inclusion criteria

1. At least 65 years old.
2. Before enrolling in the study, all participants signed an informed consent form.

### Exclusion criteria

1. Severe mental illness or neurological disorder and language-related communication difficulties.
2. Severe conditions, such as generalized inflammation or end-stage cancer.
3. Wholly disabled .

Shandong Provincial Hospital Affiliated to Shandong First Medical University Ethics Committee approved the study. All methods were performed following relevant guidelines and regulations.

### Demographic characteristics

As part of the assessment, researchers tested the frailty of the patients. They also reviewed the patient’s records to assess other characteristics (age, gender, BMI, diet, allergic history, with catheters, damaged skin, consciousness, sleep, vision, hearing, language communication, complicating disease, falls after admission, hospital admission, and hospitalisation times).

### Geriatric syndrome

1. **Pain assessment**. The Numerical Rating Scale (NRS) was used to measure the severity of pain. This scale uses 11-point marked 0 for none to 10 for the greatest pain imagina ble. The scale is valid and reliable [22].
2. **Nutritional assessment**. The Mini Nutrition Assessment Short-Form (MNA-SF) was used to assess nutritional status [23]. It contains six components. We calculated a cumulative score and divided patients into three grades: malnourished (0–7 points), at risk of malnutrition (8–11 points), normal (12–14 points). It is a validated screening tool for identifying malnourished or at-risk older adults.
3. **The Mini-Mental State Examination (MMSE)**. MMSE was used to assess the patient’s cognitive status. It contains registration, recall, attention and calculation, language, and orientation [24], a score ≥ 24 is considered normal and < 24 is defined as cognitive impairment.
4. **Depression assessment**. Geriatric Depression Scale (GDS-15) includes 15 areas of mood, life satisfaction, helplessness, energy levels, and hopelessness with Yes/No response, with a clinical threshold ≥ 5 as depressive state and < 5 normal [25]. It is reliable and validated to diagnose major depression.
5. **Fall assessment**. We performed a fall risk assessment by using the 125-point Morse Fall Scale (MFS), which encompasses questions surrounding the six areas of secondary disease, the history of falls, intravenous therapy/heparin lock, ambulatory aid, mental status, and gait. It divided patients into three grades: low (0–24 points), intermediate (25–45 points), high risk [26].
6. **Activities of daily living (ADL)**. ADL was measured by Barthel Index (BI) [27]. The total score ranges from 0 to 100 points. The higher the score, the stronger of the independence.
7. **Venous Thromboembolism (VTE) Risk Assessment**. The Caprini assessment score was used to assess the risk of VTE [28]. If the score was 0–1, patients are considered low-risk; 2 medium-risk; 3–4 high risk; ≥5 very high-risk .
8. **Braden Scale**. The Braden Scale is a valid instrument for predicting pressure ulcer risk [29]. This scale assesses six sub-scores: sensory perception, moisture, activity, mobility, nutrition, friction, and shear. Scores between 15 and 18 indicate low risk, between 13 and 14 indicate intermediate risk, 10–12 is high risk, and ≤ 9 is very high risk.
9. **Water Swallow Test**. In this study, swallowing function was assessed by the water swallowing test [30]. The time, drinking 30 ml of warm water, and cough status was observed. There are five levels based on points scored. Levels 3–5 indicate dysphagia, while levels 1–2 reflect normal swallowing.
10. **The International Consultation on Incontinence Questionnaire-Short Form (ICI-Q-SF)**. A four-grade scale was used to evaluate patients’ urinary incontinence: asymptomatic, mild, moderate, and severe, according to ICI-Q-SF [31]. No urinary incontinence (0 points), mild urinary incontinence (1–7 points), moderate urinary incontinence ( 8–14 points), and severe urinary incontinence (15–21 points).

### Frailty assessment

The assessment of frailty was conducted using the 5-item FRAIL scale, which includes items related to fatigue, resistance, ambulation, illnesses, and loss of weight, with each criterion rated as either 1 or 0 point based on the presence of specific characteristics. Scores on the FRAIL scale range from 0 to 5 points, with higher scores indicating a greater degree of frailty [32]. In this study, patients were categorized into two groups(0–2 points = non-Frailty, 3–5 points = Frailty) based on the FRAIL scale, which originally assigned 3–5 points to identify frail patients, 1–2 points to indicate prefrail status, and 0 points to correspond to robust health [33]. The FRAIL scale is valid for the older Chinese adult population [34]. Table 1 showed the components of FRAIL Scale.

**Table 1:** The FRAIL Scale

Item	Scale
Fatigue	Tired all or most of the time during the past four weeks(No/Yes)
Resistance	Difficulty walking up 10 steps without resting or aids(No/Yes)
Ambulation	Difficulty walking several hundred yards alone without aid(500–600 m)(No/Yes)
Illnesses	5 or more illness(No/Yes)
Loss of weight	Weight loss > 5% within the past year(No/Yes)

### Data collection

Geriatric nurses are authorized to assess frailty and geriatric syndrome only after obtaining consent and signature from older inpatients or their family members, and completing CGA training and acquiring relevant qualifications. Face-to-face data were collected using standardized guidance through an information personal digital assistant (PDA). Demographics and CGA were performed on patients admitted for the first time during the study period, and follow-up visits were made in person or by phone 3 months later to assess frailty.

### Statistical methods

SPSS Version 25.0 statistical software and R (version 4.1.1) were used for data processing, with non-normally distributed variables reported using medians (25th–75th percentile). Enumeration data were expressed as percentages. The Chi-square or Fisher exact test was used for intergroup comparison. All statistical tests were bilateral. Significant variables were employed in constructing risk models to predict frailty through logistic regression analysis using enter methods. The nomogram prediction model was meticulously developed utilizing the Frailty score and independent clinical factors, employing the R package rms. Nomogram performance was internally validated with Bootstrap resampling (1000 times) analysis. The final model was externally validated using an independent validation data set. A receiver operating characteristic (ROC) curve was drawn for each model. In addition, the area under the curve (AUC) of the model was calculated to evaluate the degree of separability. The AUC greater than 0.7 indicates a better classification capability for the model [35]. The calibration of the models was assessed by calibration curves. Differences were considered statistically significant at p < 0.05.

## Results

### General characteristics

Two thousand two hundred twenty-six inpatients, including 1528 men and 698 women with ages ranging from 65 to 101 [median = 74.00 (71.00, 79.00)], were initially included. Five hundred sixty-two older inpatients (25.25%) suffered from frailty; 342 (23.09%) and 220 (29.53%) comprised the modelling and validation sets, respectively.

### Analysis of frailty determinants for older inpatients

In the modelling set, demographic characteristics and geriatric syndrome factors were analyzed by univariate analysis. Table 2 presents statistically significant differences. Twenty-three significant factors in the univariate analysis were entered into logistic regression analysis with the enter method. Of these, 10 independently statistically significant predictors of frailty were included in the prediction model. These predictors included: damaged skin (OR 10.702; 95% CI1.837-62.358), MNA-SF risk of malnutrition score (OR 2.302; 95% CI 1.543–3.433 ), malnutrition (OR 3.417; 95% CI 1.703–6.855), GDS-15 score (OR 4.298; 95% CI 2.269–8.144), Morse intermediate-risk (OR 2.635; 95% CI 1.087–6.390), and high-risk (OR 4.472; 95%CI 1.729–11.566) scores, hospital admission (OR 1.776; 95% CI 1.130–2.792), ICI-Q-SF (OR 1.602; 95% CI 1.096–2.342), Braden (OR 1.741; 95% CI 1.024–2.962), MMSE (OR 1.520; 95% CI 1.055–2.191), BI mild (OR 4.375; 95% CI 2.613–7.326), moderate (OR 7.532; 95% CI 3.734–15.192), and severe dependence (OR 8.663; 95% CI 3.617–20.747), and Caprini scores (OR 1.840; 95% CI 1.180–2.871) (see Table 3).

**Table 2:** Comparison of frailty prevalence by demographics characteristics and geriatric syndrome in the modelling set

Characteristics	Cases (n = 1481)	Non-frailty (n = 1139)	Frailty (n = 342)	Statistics	P value
Cerebrovascular disease (%)]				χ2 = 6.38	0.012
Yes	169(11.41%)	143(84.62%)	26(15.38%)		
No	1312(88.59%)	996(75.91%)	316(24.09%)		
Diet				χ2 = 35.59	< 0.001
Normal diet	1086(73.33%)	878(80.85%)	208(19.15%)		
Abnormal diet	395(26.67%)	261(66.08%)	134(33.92%)		
Allergic history				χ2 = 5.36	0.021
Yes	239(16.14%)	170(71.13%)	69(28.87%)		
No	1242(83.86%)	969(78.02%)	273(21.98%)		
With catheter				χ2 = 52.56	< 0.001
Yes	93(6.28%)	43(46.24%)	50(53.76%)		
No	1388(93.72%)	1096(78.96%)	292(21.04%)		
Damaged skin				χ2 = 27.75	< 0.001
Yes	14(0.95%)	2(14.29%)	12(85.71%)		
No	1467(99.05%)	1137(77.51%)	330(22.49%)		
Consciousness				χ2 = 4.74	0.029
Yes	1457(98.38%)	1125(77.21%)	332(22.79%)		
No	24(1.62%)	14(58.33%)	10(41.67%)		
Normal sleep				χ2 = 34.07	< 0.001
Yes	1395(94.19%)	1095(78.49%)	300(21.51%)		
No	86(5.81%)	44(51.16%)	42(48.84%)		
Normal vision				χ2 = 39.12	< 0.001
Yes	1371(92.57%)	1081(78.85%)	290(21.15%)		
No	110(7.43%)	58(52.73%)	52(47.27%)		
Normal hearing				χ2 = 77.63	< 0.001
Yes	1290(87.10%)	1040(80.62%)	250(19.38%)		
No	191(12.90%)	99(51.83%)	92(48.17%)		
GDS − 15 score				χ2 = 132.20	< 0.001
Normal	1139(76.91%)	1117(98.07%)	22(1.93%)		
Abnormal	342(23.09%)	279(81.58%)	63(18.42%)		
Admission to hospital				χ2 = 330.50	< 0.001
Walking	1214(81.97%)	1047(86.24%)	167(13.76%)		
Wheelchair or stretcher	267(18.03%)	92(34.46%)	175(65.54%)		
MNA–SF score				χ2 = 196.01	< 0.001
Normal	1122(75.76%)	954(85.03%)	168(14.97%)		
Risk of malnutrition	272(18.37%)	157(57.72%)	115(42.28%)		
Malnutrition	87(5.87%)	28(32.18%)	59(67.82%)		
MFS score				χ2 = 276.98	< 0.001
Low risk	229(15.46%)	222(96.94%)	7(3.06%)		
Medium risk	946(63.88%)	787(83.19%)	159(16.81%)		
High risk	306(20.66%)	130(42.48%)	176(57.52%)		
Braden score				χ2 = 315.55	< 0.001
Without risk	1139(76.91%)	1087(95.43%)	52(4.57%)		
With risk(low or medium or high or very high)	342(23.09%)	200(58.48%)	142(41.52%)		
BI score				χ2 = 496.89	< 0.001
No dependence	714(48.21%)	690(96.64%)	24(3.36%)		
Mild dependence	503(33.96%)	370(73.56%)	133(26.44%)		
Moderate dependence	136(9.18%)	51(37.50%)	85(62.50%)		
Severe dependence	128(8.64%)	28(21.88%)	100(78.13%)		
Water swallow test				χ2 = 123.63	< 0.001
Normal	1058(71.44%)	894(84.50%)	164(15.50%)		
Abnormal	367(24.78%)	218(59.40%)	149(40.60%)		
Dysphagia	56(3.78%)	27(48.21%)	29(51.79%)		
Caprini score				χ2 = 111.68	< 0.001
Low or medium risk	1139(76.91%)	505(44.34%)	634(55.66%)		
High or very high risk	342(23.09%)	44(12.87%)	298(87.13%)		
ICI-Q-SF score				χ2 = 107.93	< 0.001
Normal	1139(76.91%)	965(84.72%)	174(15.28%)		
Mild or moderate or severe urinary incontinence	342(23.09%)	200(58.48%)	142(41.52%)		
Pain				χ2 = 14.21	< 0.001
Painless	1139(76.91%)	1083(95.08%)	56(4.92%)		
Mild or moderate or severe pain	342(23.09%)	306(89.47%)	36(10.53%)		
Age				χ2 = 205.82	< 0.001
65–74	856(57.80%)	743(86.80%)	113(13.20%)		
75–84	450(30.38%)	331(73.56%)	119(26.44%)		
≥ 85	175(11.82%)	65(37.14%)	110(62.86%)		
MMSE score				χ2 = 81.45	< 0.001
Normal	1139(76.91%)	820(71.99%)	319(28.01%)		
Abnormal	342(23.09%)	156(45.61%)	186(54.39%)		
BMI				χ2 = 49.23	< 0.001
< 18.5	88(5.94%)	49(55.68%)	39(44.32%)		
18.5–23.9	714(48.21%)	519(72.69%)	195(27.31%)		
≥ 24	679(45.85%)	571(84.09%)	108(15.91%)		
Hospital days				χ2 = 71.99	< 0.001
≤ 5	446(30.11%)	389(87.22%)	57(12.78%)		
6–9	413(27.89%)	329(79.66%)	84(20.34%)		
10–13	314(21.20%)	232(73.89%)	82(26.11%)		
≥ 14	308(20.80%)	189(61.36%)	119(38.64%)		
Hospitalisation times				χ2 = 28.68	< 0.001
1	713(48.14%)	588(82.47%)	125(17.53%)		
2	210(14.18%)	162(77.14%)	48(22.86%)		
≥ 3	558(37.68%)	389(69.71%)	169(30.29%)		
Year				χ2 = 10.89	0.001
2019–2020	1481(66.53%)	342(23.09%)	1139(76.91%)		
2021	745(33.47%)	220(29.53%)	525(70.47%)		

**Table 3:** Multivariate logistic regression analysis of frailty in hospitalised older patients

Variables	β value	SE value	OR(95%CI)	P
Damaged skin	2.370	0.899	10.702(1.837–62.358)	0.008
MNA–SF score(Normal nutritional status as control)				
Risk of malnutrition	0.834	0.204	2.302(1.543–3.433)	< 0.001
Malnutrition	1.229	0.355	3.417(1.703–6.855)	0.001
GDS-15 abmormal	1.458	0.326	4.298(2.269–8.144)	< 0.001
Morse score (Low-risk as control)				
Intermediate-risk	0.969	0.452	2.635(1.087–6.390)	0.032
High-risk	1.498	0.485	4.472(1.729–11.566)	0.002
Admission to hospital(Walk as control)	0.575	0.231	1.776(1.130–2.792)	0.013
ICI-Q-SF score Mild or moderate or severe urinary incontinence	0.471	0.194	1.602(1.096–2.342)	0.015
Braden score Low or medium or high or very high risk(No risk as control)	0.555	0.271	1.741(1.024–2.962)	0.041
MMSE score abnormal	0.419	0.186	1.520(1.055–2.191)	0.025
Barthel index score(No dependence as control)				
Mild dependence	1.476	0.263	4.375(2.613–7.326)	< 0.001
Moderate dependence	2.019	0.358	7.532(3.734–15.192)	< 0.001
Severe dependence	2.159	0.446	8.663(3.617–20.747)	< 0.001
Caprini score High or very high risk(Low or medium risk as control)	0.610	0.227	1.840(1.180–2.871)	0.007

### Development of a predictive frailty nomogram for older inpatients

**Fig. 2:**
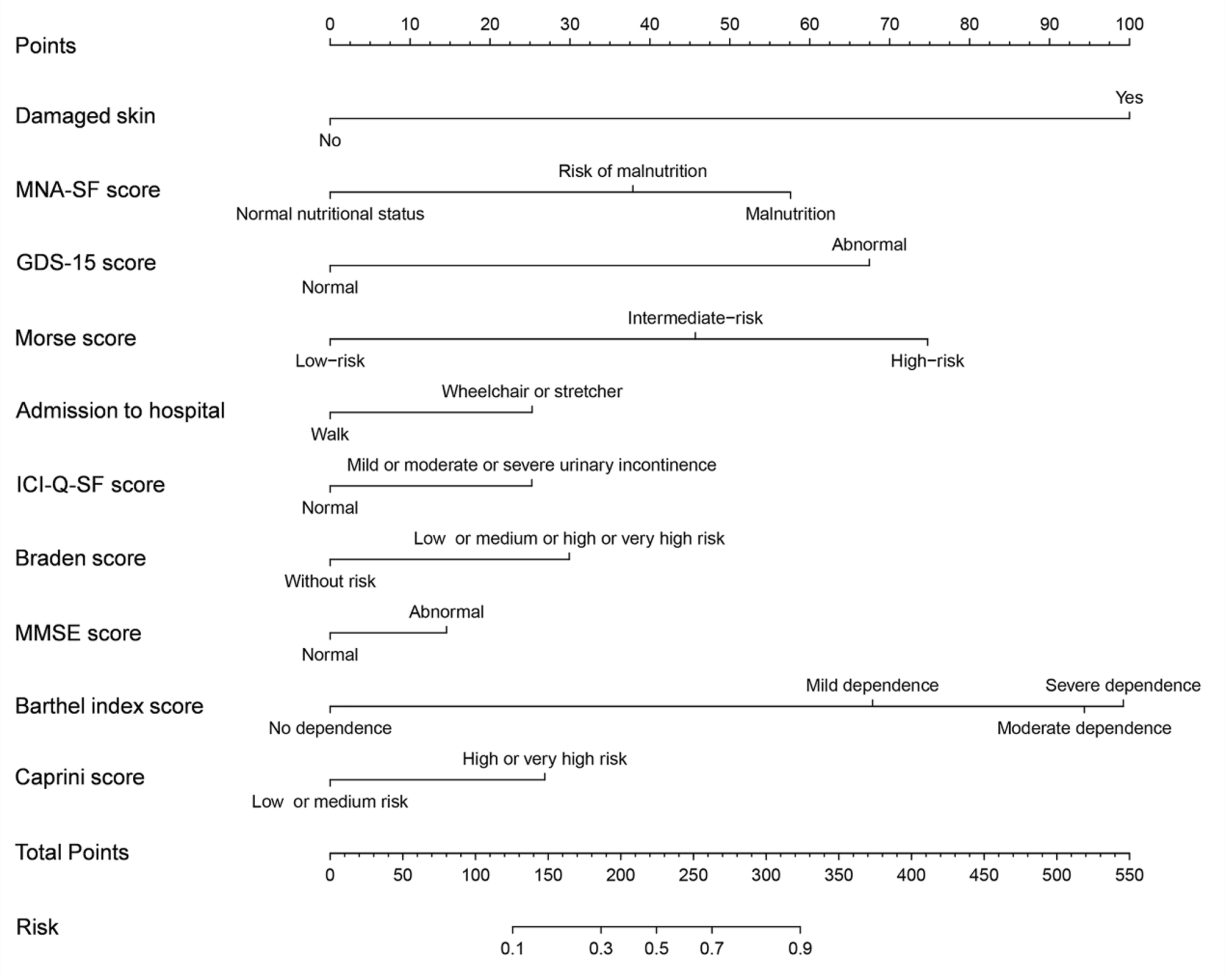
Nomogram model for frailty risk in older inpatients in training set

Combined with the independent significant factors selected in logistic regression analysis, a prediction nomogram (Fig. 2) is developed. We calculated the score for each independent risk factor by running a line from it to the scores axis. The integral sum is located on the total score axis. The probability of frailty development lies at the score where the line pulls down to the risk axis.

### Prediction of nomogram performance

A ROC curve (Figure ​3) assessed nomogram accuracy. The AUC of the nomogram was 0.910 (95%CI: 0.893–0.927).

**Fig. 3:**
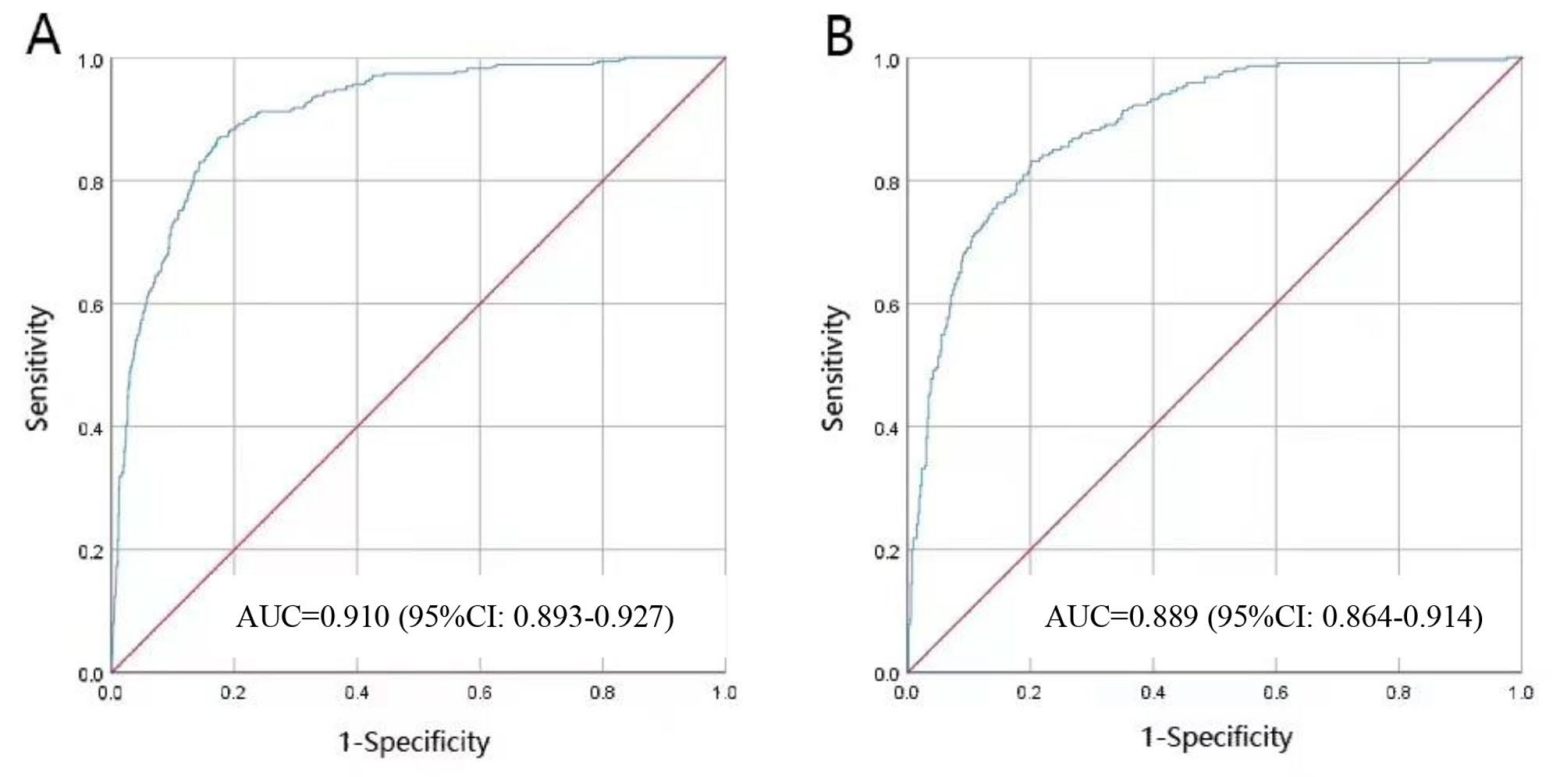
The ROC curve and AUC of the nomogram in the training set(**A**) and validation set(**B**)

The nomogram was externally validated based on 745 older inpatients from the validation set. Figure ​3B showed The ROC curve of the nomogram with external validation. The AUC of the nomogram was 0.889 (95%CI: 0.864–0.914). In the training (Fig. 3A) and validation (Fig. 3B) sets, the possibility of frailty AUC in the training (0.910) and validation (0.889) sets were adequately predictive; sufficient to identify older inpatients at risk of frailty.

**Fig. 4:**
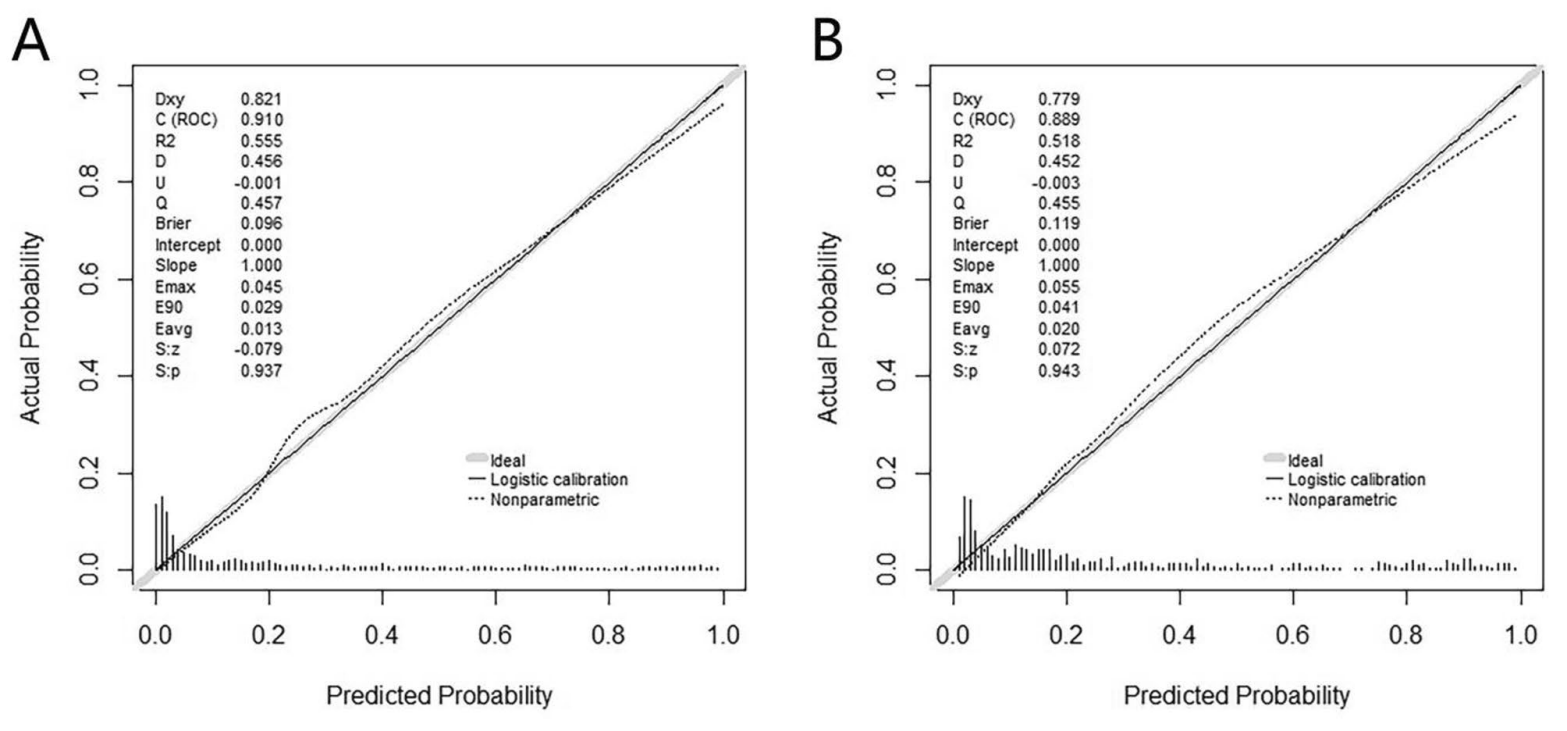
Calibration of the nomogram for the training set(**A**) and validation set(**B**)

The nomogram was internally validated by Bootstrap resampling (1000 times) and calibration plots. Figure 4 A and 4B present the calibration plots in the training and validation sets, respectively. These figures showed the concordance of the nomogram predictions. The calibration curves showed that the probability of frailty predicted by the nomogram is satisfactorily matched.

## Discussion

This study showed frailty prevalence in hospitalised older people is 25.25%, consistent with previous studies’ results estimating a range between 18 and 80% [1, 36]. Frailty progression leads to adverse outcomes, such as functional disability, hospitalisation, and death in older people. Predicting frailty risk can improve clinical decision-making and intervention. Therefore, constructing a risk prediction model to identify frailty early and intervene promptly is of great significance. In this study, we have developed and internally and externally validated a visualization nomogram prediction model for frailty in hospitalised older people using structured CGA data collected using PDA. The model displayed fair discrimination and good calibration in frailty risk prediction with an AUC of 0.910 (95%CI:0.893–0.927). The final prediction model included damaged skin, MNA-SF, GDS-15, and Morse scores, hospital admission, ICI-Q-SF, Braden, MMSE, Barthel index, and Caprini scores by logistic regression analysis. Furthermore, our model is easily applied and accessible in clinical practice. It is inexpensive and contains variables routinely collected and readily available by consultation.

The medical staff calculated the total score of 10 predictors according to the nomogram to obtain the risk prediction value of frailty in hospitalised older people. Distinguishing high- and low-risk populations according to the optimal threshold of 0.2044 can screen for frailty early and avoid medical resource waste. For example, a hospitalised older person with damaged skin (100 points), very high-risk Braden score (30 points), malnutrition (58 points), GDS-15 score normal (0 points), hospital admission by stretcher (25 points), no incontinence (0 points), MMSE score normal (0 points), ADL severe dependence (99 points), and low-risk Caprini score (0 points) can receive timely appropriate services. The total score of the nomogram model = 100 + 30 + 58 + 0 + 25 + 0 + 14 + 99 + 0 = 312 corresponds to a frailty risk probability of 0.87. This score places the individual in the high-risk group. Intervention should occur as soon as possible.

Some similarities exist between the predictors retained in our prediction model and those described in others developed using older-based research queues. Our results are consistent with those of Timothy et al. [37–44]. They found that fall risk, malnutrition, depression, Caprini score, ADL, urinary incontinence, Braden Scale, and cognitive decline are important predictors of frailty.

Our univariate analysis suggested that age, abnormal sleep, dysphagia, pain, BMI, and hospital days were risk factors for frailty. However, neither factor was an independent risk factor for frailty based on multivariable logistic regression analysis. This finding was unexpected. The possible explanation was that these analyses could not account for all possible confounders. Furthermore, a spurious or indirect association exists between those factors and frailty. More longitudinal studies are needed to clarify the roles of age, abnormal sleep, dysphagia, pain, BMI, and hospital days in old adult frailty.

This nomogram showed that the AUC was 0.910 (95%CI: 0.893–0.927) and suggested a relatively good discriminative capacity. Moreover, calibration curves, quantifying how close predictions were to the actual outcome, presented satisfactory calibration. The present study’s duration was three years. In total, 2,226 patients were enrolled, and 24 variables related to older inpatients were analyzed. We developed a new model to predict clinical frailty for hospitalised older people. Our novel prediction model is externally validated in a separate time validation queue. To sum up, we consider our frailty risk prediction model to be convenient, economical and reliable. Therefore, this method is worth popularizing in clinical practice.

### Limitations

However, our study still had some limitations. First, due to the retrospective nature of this study, selection bias may exist. Factors such as serological indicators and mobility assessment tests were not included in the study content. It is still necessary to conduct prospective randomized controlled trials to demonstrate the feasibility of intervention frailty and follow-up plans according to risk levels. Second, we developed a prediction and not a causal model. Therefore, the inclusion of other predictors significantly correlated with frailty does not necessarily lead to predictive improvements. Third, sample of damaged skin group was not large enough to obtain more precise results. In subsequent investigations, a larger cohort of elderly individuals exhibiting skin damage will be recruited to provide additional evidence regarding the influence of damaged skin on frailty. Fourth, we only followed up patients’ frailty after 3 months. In future studies, we will follow up patients’ frailty after 6 months or more to form a more scientific frailty risk prediction model and frailty trajectory.

## Conclusions

The nomogram constructed using damaged skin, MNA-SF, GDS-15, and Morse scores, hospital admission, ICI-Q-SF, Braden, MMSE, BI, and Caprini scores can predict frailty risk of hospitalised older people aged 60 or above. This nomogram showed good discrimination and calibration. Therefore, It is inexpensive, easily applied, and accessible in clinical practice, containing variables routinely collected and readily available through consultation. It will be valuable for grasp older inpatients at high risk of frailty and risk factors in hospital setting to guide the formulation of intervention measures precisely for reversing and preventing frailty.

## Data Availability

The datasets used and/or analyzed during the current study are available from the corresponding author on reasonable request.

## Abbreviations

CGA: Comprehensive geriatric assessment
BMI: Body mass index
MNA-SF: Mini Nutrition Assessment Short-Form
MMSE: Mini-Mental State Examination
MFS: Morse Fall Scale
ADL: Activities of daily living
BI: Barthel Index
ICI-Q-SF: Incontinence Questionnaire-Short Form
PDA: Personal digital assistant
ROC: Receiver operating characteristic
AUC: Area under the curve
CI: Confidence interval
OR: Odds ratio
